# Guillain-Barre Syndrome With Concomitant Severe Preeclampsia: A Case Report

**DOI:** 10.7759/cureus.40796

**Published:** 2023-06-22

**Authors:** Ronald M Swonger, Alina Syros, Lindsey Finch, Jessica Moore, Adolphia Lauture, Alvaro Soto Rincon, Nicholas Tinker, Reine Zbeidy, Labib Ghulmiyyah, Michael Paidas

**Affiliations:** 1 Miller School of Medicine, University of Miami, Miami, USA; 2 Obstetrics and Gynecology, University of Miami/Jackson Memorial Hospital, Miami, USA; 3 Obstetrics and Gynecology, Mt. Sinai Hospital, Miami, USA; 4 Anesthesiology, University of Miami/Jackson Memorial Hospital, Miami, USA; 5 Anesthesiology and Perioperative Medicine, University of Miami/Jackson Memorial Hospital, Miami, USA; 6 Obstetrics and Gynaecology, University of Miami/Jackson Memorial Hospital, Miami, USA

**Keywords:** maternal and fetal risk, gestational hypertension, pregnancy, preeclampsia, guillain-barre syndrome

## Abstract

With an estimated 100,000 new cases yearly worldwide, Guillain-Barre syndrome (GBS) is the most common cause of flaccid paralysis. GBS is exceedingly rare in pregnancy and carries high maternal and fetal risk. We report a case of a 38-year-old essential primigravida who presented at 38 weeks six days gestational age with ascending paraplegia progressing to dysarthria, dysphagia, and facial weakness. A clinical diagnosis of GBS was made in an outside institution, supported by elevated protein on lumbar puncture. During the antepartum period, a diagnosis of gestational hypertension progressed to preeclampsia with severe features when a sudden rise in liver function tests occurred. The patient underwent an uneventful planned cesarean delivery but could not be extubated due to respiratory failure. After a 20-day critical care admission, she was extubated and had an improvement in neurologic status to near her baseline.

## Introduction

Guillain-Barre syndrome (GBS) is the most common cause of flaccid paralysis, with an estimated 100,000 new cases per year worldwide [1]. It is a progressive neurologic disease that typically presents with symmetrical weakness and paresthesia of the distal lower extremities that ascends and can ultimately result in respiratory failure if left untreated [1-3]. GBS is often preceded by an infection, frequently with Campylobacter jejuni [1, 2]. When GBS is diagnosed, supportive care is the mainstay of treatment, with considerations given for intravenous immune globulin (IVIG) and plasma exchange. Plasma exchange in appropriately selected patients may prevent permanent nerve damage and respiratory failure [4]. Although rare during pregnancy, GBS is associated with high maternal and perinatal morbidity and mortality [5]. The mortality rate has been reported to range between 7% and 25% in patients with pregnancy compared to 3% to 13% in patients with no pregnancy[5].

Our patient presented from a referring hospital with the diagnosis of GBS at 38 weeks and six days gestation. During her hospital course, she developed elevated blood pressure and liver enzyme function tests (LFTs) twice the upper limit of normal, concerning preeclampsia with severe features. There is a dearth of literature describing cases involving pregnant women diagnosed with GBS with concomitant preeclampsia with severe features.

## Case presentation

A 38-year-old primigravida at 38.6 weeks gestational age (WGA) presented to our hospital as a transfer of care for the management of GBS. Her prenatal history had previously been unremarkable. At 31.2 weeks, she received her tetanus, diphtheria, and pertussis (TdAP) and influenza vaccinations and reported that she subsequently developed a flu-like illness for three days that self-resolved. Five weeks later, she began to feel bilateral tingling and numbness in her upper and lower extremities followed by progressive leg weakness. Her weakness progressed to the point that she became unable to walk or support herself. Four days later, she developed left facial weakness with mild dysphagia, dysarthria, and right facial weakness following shortly thereafter. No associated respiratory or cardiovascular symptoms were present at that time. Imaging studies were unremarkable, but cerebrospinal fluid analysis showed albuminocytologic dissociation with elevated protein of ­­­172, polymorphonuclear cells 7, red blood cells 0, glucose 54, consistent with GBS, and IVIG was initiated at 0.4/kg per day for five days. 

Initial diagnosis/assessment

Examination revealed areflexia of the lower extremities, hyporeflexia of the upper extremities, and flaccid paralysis. Hours after admission, the patient was noticed to have mild dysphagia and dysarthria followed by right facial weakness. Respiration and mentation were intact. No shortness of breath, bowel/bladder incontinence, or autonomic symptoms were reported. Her speech was fluent but slightly dysarthric. Her facial sensation was intact, but she could not close both eyes or smile. She had decreased muscle strength of 3/5, mainly in the lower extremities, and a moderate bilateral upper extremities weakness of 4/5. Light touch sensation was preserved, but subjective sensation to pinprick was diminished below mid-thigh, and proprioception was completely absent.

She was afebrile, saturating 99% without ventilatory support, but was noted to have an elevated blood pressure of 143/78. Per the record review, the patient had one additional mild-range blood pressure during her admission to the hospital before the transfer, and she was diagnosed with gestational hypertension. Labs to evaluate for preeclampsia were performed and were notable for an elevated urine protein/creatinine ratio of 0.31, and LFTs twice the upper limit of normal with aspartate aminotransferase (AST) of 161 and alanine transaminase (ALT) of 202. Based on these laboratory abnormalities, the patient was diagnosed with preeclampsia with questionable severe features due to elevated LFTs in the setting of IVIG use. A multidisciplinary meeting to discuss management options was held and included neurology, obstetrics, pediatrics, and anesthesia. The decision for cesarean delivery was made due to the worsening maternal condition, including hoarseness and dysphonia in the setting of preeclampsia with possible severe features. She was given antiemetic prophylaxis along with sodium citrate. However, she was not started on medication for seizure prophylaxis at this time, given the uncertain presence of severe features.

Standard American Society of Anesthesiology (ASA) monitors were applied. Two large-bore intravenous lines and an arterial line were placed. A modified combined spinal epidural was performed in the lateral position at the level of L3-L4, using 1.2 mL of bupivacaine 0.75% with 15 mcg of fentanyl and 0.1 mg of Duramorph in the intrathecal space. A T6 anesthetic level was slowly achieved using the epidural catheter to prevent profound hemodynamic changes. Supplemental oxygen was given through a nasal cannula, and a phenylephrine 20 mcg/minute infusion was started to prevent hypotension. The patient remained stable throughout the surgery.

Cesarean delivery was uncomplicated, and the patient delivered a male infant weighing 2,910 g, with an Apgar score of 9/9/9 and an estimated blood loss of 600 mL. Following delivery, the patient was transferred to the stepdown intensive care unit (ICU) where she was managed with Keppra 500 mg twice a day (BID) for 24 hours for seizure prophylaxis. Magnesium sulfate was avoided due to potential respiratory compromise. She received two additional IVIG treatments for a total of five. Her blood pressure remained mildly elevated, and her transaminitis showed a downward trend but did not fully normalize, with an AST level of 67 and an ALT level of 79. Despite the ongoing administration of IVIG, a formal diagnosis of preeclampsia with severe features was established due to the persistent downward trend of LFTs postpartum. Magnesium was still avoided at this time. She continued to report dysarthria and dysphagia postoperatively but had improved sensations in the lower extremities. She retained good respiratory function saturating 100% on room air postoperatively.

The patient was downgraded from the ICU to the intermediate medical care unit (IMCU) on postoperative day 2 and continued to meet postoperative milestones. She was then transferred to the internal medicine inpatient team, then to the physical medicine and rehabilitation center. Throughout her hospital stay, negative inspiratory force and vital capacity measurements were conducted to monitor her respiratory status. Additionally, daily venous blood gases were obtained to assess her respiratory function. Vital capacity was 2.5 at baseline and remained stable. She did not need respiratory support. At the time of discharge, she had persistent decreased sensation in her bilateral lower extremities and bilateral paresthesia in her upper extremities.

Her motor function is progressively improving, and her speech has normalized. She is using a walker and continuing her physical rehabilitation therapies. She does not have any residual respiratory compromise.

## Discussion

While GBS in pregnancy is rare, it is important to diagnose and treat it promptly to reduce the significant risk of morbidity and mortality. This syndrome in pregnancy has an estimated incidence between 1.2 and 1.9 cases per 100,000 people annually [6]. Association with an increased need for ventilator support and maternal mortality makes anesthesia management paramount to improve patient outcomes. Peripheral nerve demyelination can be triggered by infection or less commonly by surgery and rarely by vaccination [7].

Cases of GBS in pregnancy without concomitant preeclampsia with severe features have been described and are listed in Appendix. However, to the authors’ knowledge, this is the first case report to document GBS in pregnancy with concomitant preeclampsia with severe features. Preeclampsia is a hypertensive disorder of pregnancy that typically presents with new-onset hypertension and proteinuria after 20 weeks of gestation [8]. Preeclampsia is an increasingly common disorder and is a major cause of maternal and fetal morbidity and mortality [9].

Our patient developed symptoms of GBS four weeks after receiving the TdAP and influenza vaccinations. Although GBS is most often preceded by infectious etiologies, it has been associated with other antecedent events, including vaccinations [1,10]. In 1976, the National Influenza Immunization Program was suspended because of an increase in the reports of GBS symptoms following influenza vaccination [10]. An epidemiological study of these reports found an eightfold increased risk of developing GBS after receiving the influenza vaccination that year [11]. Since then, there have been additional studies that have shown an increased risk of GBS following seasonal influenza vaccination [11]. When the TdAP vaccination was approved for use in 2005, adverse effects, including GBS, were monitored [12]. GBS after the TdAP vaccination was compared to the background risk of GBS, and they found a relative risk of 1.60 [12]. Based on the available evidence, it was determined with relative confidence that the TdAP vaccination carries a risk of no more than approximately 1 additional case per 100,000 doses [12]. In light of the safety profile and benefits to the fetus, the American College of Obstetricians and Gynecologists (ACOG) recommends that all pregnant women receive the TdAP vaccination during each pregnancy [13]. ACOG also recommends that women who are or will be pregnant during influenza season should receive the annual influenza vaccination [13].

GBS is generally treated with a five-day course of IVIG [14]. The treatment does not differ in pregnant versus nonpregnant patients [15]. For non-obstetrical-related conditions, IVIG is recommended in pregnant patients as indicated for nonpregnant patients [15]. Another option for treatment includes plasma exchange (200-250 mL/kg) for five sessions but was deferred in favor of IVIG in our case [14]. Our patient’s course was further complicated by her new diagnosis of preeclampsia with possible severe features based on mild-range blood pressures and lab abnormalities, including LFTs twice the upper limit of normal and new-onset proteinuria. Despite the potential for IVIG therapy to induce transaminitis, the decision to proceed with delivery was made based on the presence of preeclampsia beyond 37 weeks of gestation and the deterioration of the maternal condition in relation to her underlying GBS. ACOG recommends delivery rather than expectant management beyond 37 weeks for gestational hypertension or preeclampsia without severe features or beyond 34 weeks gestation for women with preeclampsia with severe features. While the initiation of magnesium sulfate therapy for seizure prophylaxis is recommended for preeclampsia with severe features, we did not initiate it in this case due to an unclear diagnosis of preeclampsia with severe features and due to the potential for further respiratory compromise [16]. Although controversial, at the recommendation of neurology and the ICU teams, we administered Levetiracetam for 24 hours after delivery for seizure prophylaxis.

The mode of action for magnesium sulfate in the prevention and treatment of eclamptic seizures is not clearly understood. The anticonvulsant activity may be mediated by magnesium’s role as an N-methyl-D-aspartate (NMDA) antagonist, which may prevent and control eclamptic seizures by inhibiting NMDA receptors and blocking the neuronal damage associated with ischemia. Another potential mechanism is that magnesium sulfate may induce cerebral vasodilation, resulting in a reduction of cerebral ischemia. Additionally, magnesium's calcium antagonist and smooth muscle relaxant effects could help restrict paracellular transport of vascular contents and decrease the factors that contribute to cerebral edema and seizure activity. Side effects of magnesium sulfate include flushing, muscle weakness, and nausea. Serious adverse effects such as respiratory and cardiac arrest are rare, but if they do occur are potentially life-threatening. Although respiratory muscle paralysis only occurs at magnesium serum concentrations at or greater than 7 to 8 mmol/L, magnesium sulfate was not given due to the theoretical risk of potential further respiratory compromise [17].

One of the most serious complications of GBS during anesthesia is broncho-aspiration due to bulbar dysfunction [18]. Other considerations for patients include perioperative respiratory insufficiency due to muscle weakness. If general anesthesia is considered, succinylcholine should be avoided and replaced with a nondepolarizing muscle relaxant due to an increased risk of hyperkalemia. However, the use of nondepolarizing neuromuscular blocking agents on these patients with GBS presents the risk of prolonged blockade, requiring ventilatory support postoperatively [19].

The neuraxial technique should be managed with caution due to autonomic dysfunction with possible hemodynamic instability and autonomic hyperreflexia-type reactions. Arrhythmias and cardiac arrest could also be anticipated after neuraxial blockade [20]. Another risk in these cases is venous thromboembolism, often necessitating the administration of anticoagulation therapy in patients [21].

Anesthetic management of these patients should always include a multidisciplinary approach to facilitate the best clinical outcomes intra- and postoperatively. The anesthetic technique for pregnant women with GBS requiring cesarean delivery remains at the discretion of the specialist, who should be guided by the clinical conditions and comorbidities of each patient. In our case, the patient had stable respiratory mechanics, and the decision was made to employ the neuraxial technique.

## Conclusions

GBS is exceedingly rare in pregnancy and carries high maternal and fetal risk. This case is the only case to our knowledge involving the management of GBS in a pregnant patient with a concomitant diagnosis of severe preeclampsia. In these rare cases, delivery timing should be individualized and dependent on maternal clinical status. In situations of impending respiratory failure, an emergency cesarean section is required at gestational ages at or beyond viability. Future studies are needed to examine the laboratory diagnosis of preeclampsia in circumstances with confounding factors, as a diagnostic method exclusive to preeclampsia does not exist. This investigation is even more urgent in light of similar presentations seen with SARS-CoV-2.

## Appendices

**Table 1:** Reported cases of Guillain-Barre syndrome in pregnancy.

Title	Authors	Journal/Book	Publication Year
Facial diplegia variant of Guillain-Barré syndrome in pregnancy following COVID-19 vaccination: a case report	Zubair AS, Bae JY, Desai K.	Cureus	2022
Guillain-Barré syndrome during pregnancy: a case series	Ždraljević M, Radišić V, Perić S, Kačar A, Jovanović D, Berisavac I	J Obstet Gynaecol Res	2022
Therapeutic plasma exchange in a patient with acute motor axonal neuropathy subtype of Guillain-Barre syndrome and systemic lupus erythematosus	Akgun Y, Langlie J, Huberman MA, Wu Y	J Clin Apher	2022
Acute quadriplegia in a lactating woman with mastitis and breast abscess	Ameli G, Maier JT, Abu Daher G, Mihajlov V, Hellmeyer L	J Hum Lact	2022
Guillain-Barré syndrome in pregnancy	Andrea H, Lukáš H, René J, Igor S, Ondřej H, Petr J	Ceska Gynekol	2021
Guillain-Barre syndrome in pregnancy	González-Obando P, Martínez-Gaviria JD, Peláez-Domínguez MC, Cruz-Agudelo DC, Marulanda-Uribe JD, Quintero-Acosta W	Rev Neurol	2021
Intravenous immunoglobulin in COVID-19 associated Guillain-Barré syndrome in pregnancy	Garcia JJ, Turalde CW, Bagnas MA, Anlacan VM	BMJ Case Rep	2021
Guillain Barre Syndrome following delivery in a pregnant woman infected with SARS-CoV-2	Tekin AB, Zanapalioglu U, Gulmez S, Akarsu I, Yassa M, Tug N	J Clin Neurosci	2021
A case report of Guillain-Barré syndrome in a pregnant woman infected by COVID-19	Mehrpour M, Arab M, Hadavand F, Khalafi M, Khalafi M	Acta Neurol Belg	2021
Wernicke's encephalopathy post hyperemesis gravidarum misdiagnosed as Guillain-Barre syndrome: lessons for the frontline	Kirty K, Sarda Y, Jacob A, Venugopala D	BMJ Case Rep	2021
Bilateral Facial Palsy: A Clinical Approach	Yang A, Dalal V	Cureus	2021
Miller Fisher syndrome treated with plasmapheresis during pregnancy: Case report and review of the literature	Ángel-Páez JA, Hurtado-Bugna S, Aragón-Mendoza RL, Altman-Restrepo M, Díaz-Yamal IJ, Centanaro-Meza GA	Rev Colomb Obstet Ginecol	2021
A unique association of bifacial weakness, paresthesia and vestibulocochlear neuritis as post-COVID-19 manifestation in pregnant women: a case report	Aasfara J, Hajjij A, Bensouda H, Ouhabi H, Benariba F	Pan Afr Med J	2021
Guillain-Barré syndrome during the postpartum period	Aabdi M, Mellagui Y, Bensaid A, Bkiyar H, Housni B	Cureus	2020
Paraparetic variant of Guillain-Barré syndrome in first 24 hours of postpartum period: a case report	Aljaafari D, Ishaque N	Sultan Qaboos Univ Med J	2020
Clinico-epidemiological and genomic profile of first Zika Virus outbreak in India at Jaipur city of Rajasthan state	Malhotra B, Gupta V, Sharma P, Singh R, Sharma H, Vyas M, et al.	J Infect Public Health	2020
A case of Guillain-Barre syndrome with pregnancy who delivered in ICU: a rare outcome of rare co-occurrence	Jain R, Rathi PS, Telang K, Zaidi A	BMJ Case Rep	2019
Guillain-Barré syndrome in pregnancy: Successful multidisciplinary approach	Kasaven LS, Basu C	Eur J Obstet Gynecol Reprod Biol	2018
Neuroimaging findings in normocephalic newborns with intrauterine Zika virus exposure	Mulkey SB, Vezina G, Bulas DI, Khademian Z, Blask A, Kousa Y, et al.	Pediatr Neurol	2018
Guillain-Barré syndrome in pregnancy: a case report	Hukuimwe M, Matsa TT, Gidiri MF	Womens Health (Lond)	2017
Guillain Barré syndrome and diabetic acido-ketotic decompensation during pregnancy: a case report and review of the literature	Affes L, Elleuch M, Mnif F, Kacem FH, Salah DB, Mnif M, et al.	Pan Afr Med J	2017
Anesthesia for cesarean section in parturient with Guillain Barre Syndrome: a case report	Bouslama MA, Brahim A, Kaabia O, Khlifi A, Tarmiz K, Ben Jazia K	Tunis Med	2017
Gitelman syndrome: a rare life-threatening case of hypokalemic paralysis mimicking Guillain-Barré syndrome during pregnancy and review of the literature	Elkoundi A, Kartite N, Bensghir M, Doghmi N, Lalaoui SJ	Clin Case Rep	2017
Miller-Fisher syndrome after coronary artery bypass surgery	Aldag M, Albeyoglu S, Ciloglu U, Kutlu H, Ceylan L	Cardiovasc J Afr	2017
Guillain-Barré syndrome in pregnancy: a conservatively managed case	Fernando TN, Ambanwala AM, Ranaweera P, Kaluarachchi A	J Family Med Prim Care	2016
Importance of diagnostic work-up of Guillain-Barré syndrome in pregnancy	Pollak-Christian E, Lee KS	BMJ Case Rep	2016
Guillain-Barré syndrome and cytomegalovirus infection during pregnancy	Lupo J, Germi R, Jean D, Baccard-Longère M, Casez O, Besson G, et al.	J Clin Virol	2016
[Guillain-Barré syndrome due to Zika virus during pregnancy]	Reyna-Villasmil E, López-Sánchez G, Santos-Bolívar J	Med Clin (Barc)	2016
Guillain-Barré syndrome after trivalent influenza vaccination during pregnancy	Tomimatsu T, Sugihara M, Nagai T, Sunada Y, Kimura T, Shimoya K	Eur J Obstet Gynecol Reprod Biol	2016
Local Transmission of Zika Virus--Puerto Rico, November 23, 2015-January 28, 2016	Thomas DL, Sharp TM, Torres J, Armstrong PA, Munoz-Jordan J, Ryff KR, et al.	MMWR Morb Mortal Wkly Rep	2016
Three cases of Zika virus imported in Italy: need for a clinical awareness and evidence-based knowledge	Nicastri E, Pisapia R, Corpolongo A, Fusco FM, Cicalini S, Scognamiglio P, et al.	BMC Infect Dis	2016
Zika virus infection in 18 travellers returning from Surinam and the Dominican Republic, The Netherlands, November 2015-March 2016	Duijster JW, Goorhuis A, van Genderen PJ, Visser LG, Koopmans MP, Reimerink JH, et al.	Infection	2016
A case of successful management of Guillain-Barre syndrome in pregnancy	Pradhan D, Dey S, Bhattacharyyaa P	JNMA J Nepal Med Assoc	2015
Relapsing Guillain-Barre syndrome in pregnancy and postpartum	Meenakshi-Sundaram S, Swaminathan K, Karthik SN, Bharathi S	Ann Indian Acad Neurol	2014
A rare case of Guillain-Barré syndrome in pregnancy treated with plasma exchange	Vasudev R, Raina TR	Asian J Transfus Sci	2014
Guillain-Barré syndrome in pregnancy: an unusual case	Zafar MS, Naqash MM, Bhat TA, Malik GM	J Family Med Prim Care	2013
Anesthesia for cesarean section in a patient with Guillain-Barre syndrome: case report	Volquind D, Fellini RT, Rose GL, Tarso GP	Braz J Anesthesiol	2013
Reversible conduction failure is distinct from neurophysiological patterns of recovery in mild demyelinating Guillain-Barré syndrome	Kokubun N, Shahrizaila N, Hirata K, Yuki N	J Neurol Sci	2013
Association between acute motor axonal neuropathy and ophthalmoplegia during pregnancy	Yerdelen D, Kucukgoz U, Karatas M	J Clin Neurosci	2011
Guillain-Barré syndrome in pregnancy: a rare complication of varicella	Modi M, Singla M, Aggarwal N, Singla V, Sharma A.	Taiwan J Obstet Gynecol	2010
Experience of rehabilitation for Guillain-Barré syndrome during and after pregnancy: a case study	Wada S, Kawate N, Morotomi N, Matsumiya T, Ono G, Mizuma M	Disabil Rehabil	2010
Good maternal and fetal outcomes of predominantly sensory Guillain-Barré syndrome in pregnancy after intravenous immunoglobulin	Matsuzawa Y, Sakakibara R, Shoda T, Kishi M, Ogawa E	Neurol Sci	2010
Guillain-Barré syndrome in pregnancy: early diagnosis and treatment is essential for a favorable outcome	Campos da Silva F, de Moraes Paula G, Dos Santos Esteves Automari CV, Mendes de Almeida DS, Ubirajara Cavalcanti Guimarães R	Gynecol Obstet Invest	2009
Successful maternal and fetal outcome of Guillain-Barré syndrome complicating pregnancy	Bahadur A, Gupta N, Deka D, Mittal S	Indian J Med Sci	2009
Weakness in pregnancy - expect the unexpected	Furara S, Maw M, Khan F, Powell K	Obstet Med	2008
Severe seafood poisoning in French Polynesia: a retrospective analysis of 129 medical files	Gatti C, Oelher E, Legrand AM	Toxicon	2008
Anesthetic management of Guillain-Barré syndrome in pregnancy	Kocabas S, Karaman S, Firat V, Bademkiran F	J Clin Anesth	2007
A case of Guillain-Barre syndrome in puerperium	Batashki I, Markova D, Milchev N, Uchikova E, Dimitrova E	Akush Ginekol (Sofiia)	2007
Severe Guillain-Barré syndrome and pregnancy: two cases with rapid improvement post-partum	Vaduva C, de Seze J, Volatron AC, Stojkovic T, Piechno S, Husson J, et al.	Rev Neurol (Paris)	2006
A rare case of Guillain-Barre syndrome with pregnancy	Vijayaraghavan J, Vasudevan D, Sadique N, Rajeswari KS, Pondurangi M, Jayshree	J Indian Med Assoc	2006
Guillain-Barré syndrome and pregnancy	Boughammoura-Bouatay A, Hizem Y, Chebel S, Frih-Ayed M	Rev Med Interne	2005
Repeated use of epidural anaesthesia for caesarean delivery in a patient with Guillain-Barré syndrome	Alici HA, Cesur M, Erdem AF, Gursac M	Int J Obstet Anesth	2005
Worsening of neurologic symptoms after epidural anesthesia for labor in a Guillain-Barré patient	Wiertlewski S, Magot A, Drapier S, Malinovsky JM, Péréon Y	Anesth Analg	2004
Guillain-Barre syndrome following resection of glioblastoma multiforme	Puertas-Muñoz I, Miranda-Lloret P, Lagares A, Ramos-González A	Rev Neurol	2004
Guillain-Barre syndrome during CMV infection complicating twin pregnancy--a case report	Magoń T, Obrzut B, Kluz S, Chruściel A, Skret A	Ginekol Pol	2004
Congenital Guillain-Barré syndrome associated with maternal inflammatory bowel disease is responsive to intravenous immunoglobulin	Bamford NS, Trojaborg W, Sherbany AA, De Vivo DC	Eur J Paediatr Neurol	2002
Late onset postpartum eclampsia: a rare and difficult diagnosis	Dziewas R, Stögbauer F, Freund M, Lüdemann P, Imai T, Holzapfel C, et al.	J Neurol	2002
Massive intravenous immunoglobulin treatment in pregnancy complicated by Guillain-Barré syndrome	Yamada H, Noro N, Kato EH, Ebina Y, Cho K, Fujimoto S	Eur J Obstet Gynecol Reprod Biol	2001
A pregnant woman with hepatitis A and Guillain-Barré	Breuer GS, Morali G, Finkelstein Y, Halevy J	J Clin Gastroenterol	2001
Combined spinal and epidural anesthesia for labor and cesarean delivery in a patient with Guillain-Barre syndrome	Vassiliev DV, Nystrom EU, Leicht CH	Reg Anesth Pain Med	2001
Pregnancy, anaesthesia and guillain-barre syndrome	Nesbitt I	Anaesthesia	2000
Pregnancy, anaesthesia and Guillain Barré syndrome	Brooks H, Christian AS, May AE	Anaesthesia	2000
A case of Bickerstaff's brainstem encephalitis during pregnancy	Funakawa I, Ogoshi M, Shibazaki K, Koga M, Yuki N	Rinsho Shinkeigaku	1999
Guillain-Barré syndrome and cytomegalovirus infection during pregnancy	Mendizabal JE, Bassam BA	South Med J	1997
Guillain-Barré syndrome in mother and newborn child	Luijckx GJ, Vles J, de Baets M, Buchwald B, Troost J	Lancet	1997
Landry Guillain-Barre-Strohl syndrome in pregnancy: case report	Amoah GB	East Afr Med J	1996
Landry-Guillain-Barre-Strohl syndrome in pregnancy	Yaginuma Y, Kawamura M, Ishikawa M	J Obstet Gynaecol Res	1996
Guillain-Barre syndrome. Cytomegalovirus infections and pregnancy	Bonnema SJ	Ugeskr Laeger	1996
Pregnancy complicated by Guillain-Barré syndrome	Kuller JA, Katz VL, McCoy MC, Hansen WF	South Med J	1995
Guillain-Barré syndrome in pregnancy--two case reports and a discussion on management	Bolik A, Wissel J, Rolfs A	Arch Gynecol Obstet	1995
Guillain-Barré syndrome in pregnancy: reflections on immunopathogenesis	Rolfs A, Bolik A	Acta Neurol Scand	1994
Guillain-Barré syndrome in pregnancy--an indication for caesarian section?	Rockel A, Wissel J, Rolfs A	J Perinat Med	1994
Guillain-Barre syndrome and pregnancy	Bouaggad A, Bouderka MA, Laraki M, Barrou H, Harti A, Benaguida M	Rev Fr Gynecol Obstet	1994
Guillain-Barré polyradiculitis in pregnant women and puerperae	Burshinov AO, Deev AS	Akush Ginekol (Mosk)	1994
Guillain-Barré syndrome and pregnancy	Panduro Baron JG, Gamboa R, Gaxiola Castro R, Zamora Gallegos GY, Arellano Loera D	Ginecol Obstet Mex	1993
Guillain-Barré syndrome, pregnancy, and plasmapheresis	Clifton ER	J Am Osteopath Assoc	1992
Landry Guillain-Barré Strohl syndrome in pregnancy: report of three cases treated with plasmapheresis	Hurley TJ, Brunson AD, Archer RL, Lefler SF, Quirk JG Jr.	Obstet Gynecol	1991
Intensive care management of Guillain-Barré syndrome during pregnancy	Gautier PE, Hantson P, Vekemans MC, Fievez P, Lecart C, Sindic C, et al.	Intensive Care Med	1990
Cardiac arrest after succinylcholine administration in a pregnant patient recovered from Guillain-Barré syndrome	Feldman JM	Anesthesiology	1990
The association of acute polyradiculoneuritis, transitory diabetes insipidus and pregnancy. Apropos of a case and review of the literature	Berteau P, Morvan J, Bernard AM, Verjut JP, Cléophax JP	J Gynecol Obstet Biol Reprod (Paris)	1990
Guillain-Barré syndrome in pregnancy	Laufenburg HF, Sirus SR	Am Fam Physician	1989
The Guillain-Barre syndrome in pregnancy	Gulowsen E	Tidsskr Nor Laegeforen	1989
Landry-Guillain-Barré-Strohl syndrome in pregnancy. A report of two cases	May SE, Caudle MR, Kleinman GE	J Reprod Med	1989
Guillain-Barre syndrome after obstetrical epidural analgesia	Gautier PE, Pierre PA, Van Obbergh LJ, Van Steenberge A	Reg Anesth	1989
Acute intermittent porphyria in pregnancy	Milo R, Neuman M, Klein C, Caspi E, Arlazoroff A	Obstet Gynecol	1989
Guillain-Barré syndrome in pregnancy. A case report	Quinlan DJ, Moodley J, Lalloo BC, Nathoo UG	S Afr Med J	1988
Guillain-Barré syndrome due to hypophosphatemia following intravenous hyperalimentation	Hoff SD, Rowlands BJ	JPEN J Parenter Enteral Nutr	1988
Guillain-Barré syndrome associated with cytomegalovirus infection	Hart IK, Kennedy PG	Q J Med	1988
Management of labour and delivery in a patient with Guillain-Barré syndrome	McGrady EM	Anaesthesia	1987
[Polyradiculoneuritis and pregnancy. Apropos of 1 case]	Marsepoil T, Corjon P, Levesque P, Venutolo F, Blin F	Rev Fr Gynecol Obstet	1987
Guillain-Barre syndrome in pregnancy	d'Ambrosio G, de Angelis G	Rev Neurol (Paris)	1985
Management of Landry-Guillain-Barré syndrome in pregnancy	Nelson LH, McLean WT Jr.	Obstet Gynecol	1985
Water intoxication and hyponatremic encephalopathy from the use of an oxytocin nasal spray. A case report	Seifer DB, Sandberg EC, Ueland K, Sladen RN	J Reprod Med	1985
Maternal Guillain-Barré syndrome	Edwards R	J Neurosurg Nurs	1984
Nursing care of patients with Guillain-Barré disease in the acute stage	Solognier ME	Tijdschr Ziekenverpl	1982
Obstetric management of Landry-Guillain-Barré syndrome: a case report	Bravo RH, Katz M, Inturrisi M, Cohen NH	Am J Obstet Gynecol	1982
Guillain-Barré syndrome and pregnancy	Joensen P	Ugeskr Laeger	1981
Successful treatment by plasma exchange in Guillain-Barré syndrome with immune complexes	Valbonesi M, Mosconi L, Garelli S, Zerbi D, Celano I	Vox Sang	1980
The Landry-Guillain-Barré syndrome and pregnancy	Ahlberg G, Ahlmark G	Acta Obstet Gynecol Scand	1978
Dissecting aneurysm of the anterior cerebral artery and Guillain-Barré-syndrome during pregnancy (author's transl)	Pilz P	J Neurol	1977
Guillain-Barré polyradiculitis in pregnancy	Müller E	Cesk Gynekol	1975
